# Towards Real-Time Detection of Gait Events on Different Terrains Using Time-Frequency Analysis and Peak Heuristics Algorithm

**DOI:** 10.3390/s16101634

**Published:** 2016-10-01

**Authors:** Hui Zhou, Ning Ji, Oluwarotimi Williams Samuel, Yafei Cao, Zheyi Zhao, Shixiong Chen, Guanglin Li

**Affiliations:** 1 CAS Key Laboratory of Human-Machine Intelligence-Synergy Systems, Shenzhen Institutes of Advanced Technology, Chinese Academy of Sciences (CAS), Shenzhen 518055, China; hui.zhou@siat.ac.cn (H.Z.); rincyjining@163.com (N.J.); samuel@siat.ac.cn (O.W.S.); sx.chen@siat.ac.cn (S.C.); 2 Shenzhen College of Advanced Technology, University of Chinese Academy of Sciences, Shenzhen 518055, China; 3 Shenzhen Traditional Chinese Medicine Hospital, Shenzhen 518033, China; 4 School of Data and Computer Science, Sun Yat-Sen University, Guangzhou 510006, China; charlottezhao@foxmail.com

**Keywords:** gait event detection, accelerometer, continuous wavelet transform, heuristics, stairs walking

## Abstract

Real-time detection of gait events can be applied as a reliable input to control drop foot correction devices and lower-limb prostheses. Among the different sensors used to acquire the signals associated with walking for gait event detection, the accelerometer is considered as a preferable sensor due to its convenience of use, small size, low cost, reliability, and low power consumption. Based on the acceleration signals, different algorithms have been proposed to detect toe off (TO) and heel strike (HS) gait events in previous studies. While these algorithms could achieve a relatively reasonable performance in gait event detection, they suffer from limitations such as poor real-time performance and are less reliable in the cases of up stair and down stair terrains. In this study, a new algorithm is proposed to detect the gait events on three walking terrains in real-time based on the analysis of acceleration jerk signals with a time-frequency method to obtain gait parameters, and then the determination of the peaks of jerk signals using peak heuristics. The performance of the newly proposed algorithm was evaluated with eight healthy subjects when they were walking on level ground, up stairs, and down stairs. Our experimental results showed that the mean F1 scores of the proposed algorithm were above 0.98 for HS event detection and 0.95 for TO event detection on the three terrains. This indicates that the current algorithm would be robust and accurate for gait event detection on different terrains. Findings from the current study suggest that the proposed method may be a preferable option in some applications such as drop foot correction devices and leg prostheses.

## 1. Introduction

In recent years, the demand for efficient gait event detection has been steadily increasing. Gait event identification can be used to control the on/off time of functional electrical stimulation devices for drop foot correction in stroke patients [1,2,3,4], operate an active orthotic device for ankle foot pathologies [5], assess rehabilitation effects in post-stroke patients with gait abnormality [6], and classify daily activity to aid exercise for health care in the elderly [7]. Toe off (TO) and heel strike (HS) are two key gait events commonly used to distinguish a gait cycle into either swing phase or stance phase. Signals acquired from ground reaction force (GRF) and optoelectronic motion capture have been widely used for the detection of TO and HS events in previous studies [8,9]. A major limitation of these systems is that they are expensive and restricted to a controlled laboratory environment [10]. To overcome these drawbacks, various wearable sensors have been developed for gait event detection [11]. 

Although force sensitive resistor (FSR) sensors based on foot switches have been used to detect gait events and as well control the on/off time of functional electrical stimulation systems [12,13], such FSR sensors have certain problems in real-time applications. The sensors’ don and doff procedure are usually complex, which often introduce some level of inconvenience and discomfort to the wearers. In addition, the sensors are sometimes unreliable and can only be used in limited cycles [14]. Beside the FSR sensors, wearable inertial sensors such as accelerometers and gyroscopes have also been used extensively for gait event detections in recent years [15,16,17]. Gyroscopes are usually attached to human feet or legs to measure the angular velocity from which gait events are detected. Previous studies have shown that gyroscopes are not affected by gravitational force and are also less sensitive to the sensor positions [18,19]. However, the power consumption rate of a typical gyroscope sensor is relatively large (usually several mA), which limits its long-term use in everyday life. Accelerometers are another type of widely used wearable inertial sensors with relatively low power consumption (usually a few μA), and have been shown to provide reliable measures of gait parameters [20]. Several algorithms have been proposed to detect TO and HS events from accelerometer signals in previous studies, among which the threshold-based algorithms have been proven to be reliable for gait event detection and can be used to trigger functional electrical stimulation during level ground walking [1,2]. The inflection and extreme points-based algorithm based on differential acceleration signal has shown gait event detection results with potential use in drop foot correction during level ground walking [3].

Furthermore, physical activities during stair walking have attracted more attention in recent years because stair walking is considered as a challenging locomotion activity where most fall-related accidents occur [21]. Some work has been done to improve the performance of leg prostheses during different gait phases on stairs [22,23]. Gait characteristics on stair terrains have been widely used for the assessment and diagnosis of motor function disorder as well as for the prediction of fall risks in the elderly [24,25]. Reliable detection of gait events during stair walking would aid proper gait analysis and the development of efficient assistive devices for patients with drop foot. Some recent progress made on gait even detection on stair terrains are reported as follows. Kotiadis et al. indicated that poor detection rates were often achieved with existing methods in the terrains of upstairs and downstairs compared to level ground walking [2]. Recently, Khandelwal et al. proposed a wavelet based time-frequency analysis method to detect gait events with acceleration signals recorded from real world walking environments [20]. Although their algorithm could achieve gait event detection with relatively good accuracy and robustness, it is an offline-based method that may be inapplicable in a number of real-time applications, such as the triggering of functional electrical stimulation based devices and active prostheses. It is important to note that the reliable and accurate detection of toe off and heal strike gait events from acceleration signals while walking on different terrains (level ground, up stairs, down stairs) during daily activities still remains a major challenge for real-time applications.

In this study, in an attempt to improve the performance of gait-event detection based on acceleration signals, a new algorithm that combines time-frequency analysis and a heuristic approach is proposed for the real-time detection of gait events on three walking terrains. The performance of the newly proposed method in detecting gait events was investigated with data collected from eight healthy subjects during walking on stairs (up and down) and level ground. In addition, the proposed method was compared with the commonly used FSR based method. To evaluate the performance of gait-event detection, precision, recall, F1-score, and time agreement, metrics were adopted in the study.

## 2. Materials and Methods 

### 2.1. Subjects

Eight healthy subjects (five males and three females aged 20–30 years, with 1.55–1.78 m height and 47–78 kg weight) were recruited. The recruitment of subjects and the experimental protocols were approved by the Ethics Committee for Human Research, Shenzhen Institutes of Advanced Technology, Chinese Academy of Sciences. All subjects were informed about the purpose and experimental procedure of the study.

### 2.2. Protocol

**Figure 1:**
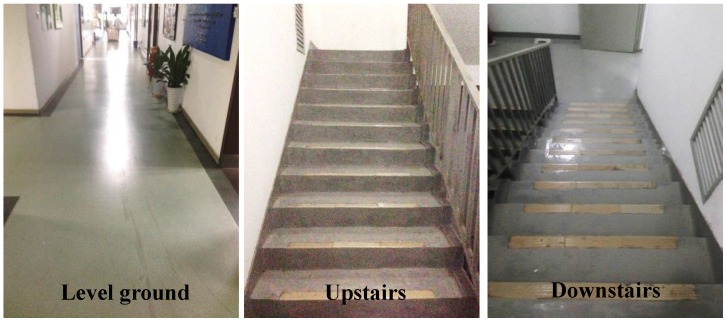
The experiment was conducted on three terrains (level ground, up stairs, and down stairs).

In the experiments, all subjects were asked to walk for about ten seconds on each of the three different terrains with a normal comfortable speed. For level ground walking, the subjects were instructed to walk along a 10 m long path in 10 s and an average walking speed of 1.5 m/s was considered in the study. The three common terrains were level ground, down stairs, and up stairs (see Figure 1). Level ground walking was tested along a long path in a straight flat corridor. The stairs consisted of 12 steps and each step was 0.15 m high and 0.25 m wide (see Figure 1). For stair walking, subjects were instructed to walk step over step and the first step was taken by the right foot. The walking experiment on each terrain was repeated 15 times (trials) per subject. 

### 2.3. Instrumentation and Data Acquisition 

**Figure 2:**
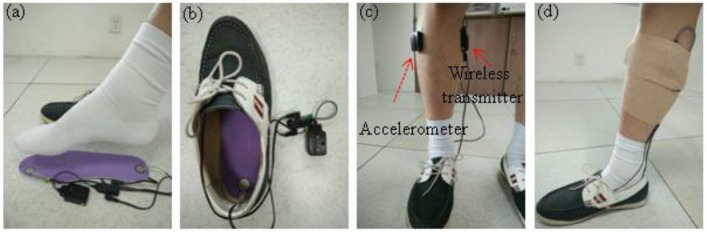
Experimental setup of force sensitive resistor (FSR) and accelerometer sensors. (**a**) The FSR sensors were mounted under the big toe and heel of the insole; (**b**) The placement of the insole into the shoe; (**c**) The placement of the accelerometer and FSR transmitter on the right leg; (**d**) A bandage was used to fix the accelerometer and FSR transmitter position.

A wireless tri-axial accelerometer sensor (Delsys Inc., Natick, MA, USA) was used to record the acceleration of the right leg during walking. In the study, the tibialis anterior (TA) muscle of the lower leg was chosen as the site for acceleration signal recording, with a consideration of possible applications of the proposed method in drop foot correction. For each subject, the skin of their TA muscle was cleaned with 75% isopropyl alcohol prior to the start of the experiment, and then the accelerometer was attached to the TA muscle with medical grade double sided adhesive tape. Additionally, a bandage (Kindmax Inc., Irvine, CA, USA) was used to fix the accelerometer to the lower limb, to minimize sensor movement and vibration during the gait (see Figure 2). For comparison purposes, the foot-switch signals were simultaneously recorded by FSR sensors (Delsys Inc., Natick, MA, USA) on the right foot. In the experiment, two FSR sensors mounted on the insole were placed under the toe and heel of each subject, as shown in Figure 2. Both the FSR and acceleration signals were wirelessly sent to the computer by a transmitter immediately after they were recorded. The sampling rate of both signals was 148.15 Hz. The 3-axis acceleration recordings were then filtered by a digital high-pass filter with a cutoff frequency of 0.5 Hz (second order Butterworth filter applied forward and backward) to reduce low frequency noises. The filtered acceleration signals were used to validate the proposed algorithm. All data were processed and analyzed in Matab R2012b (The Mathworks, Inc., Natick, MA, USA).

### 2.4. Proposed Real-Time Detection Algorithm of Gait Events

**Figure 3:**
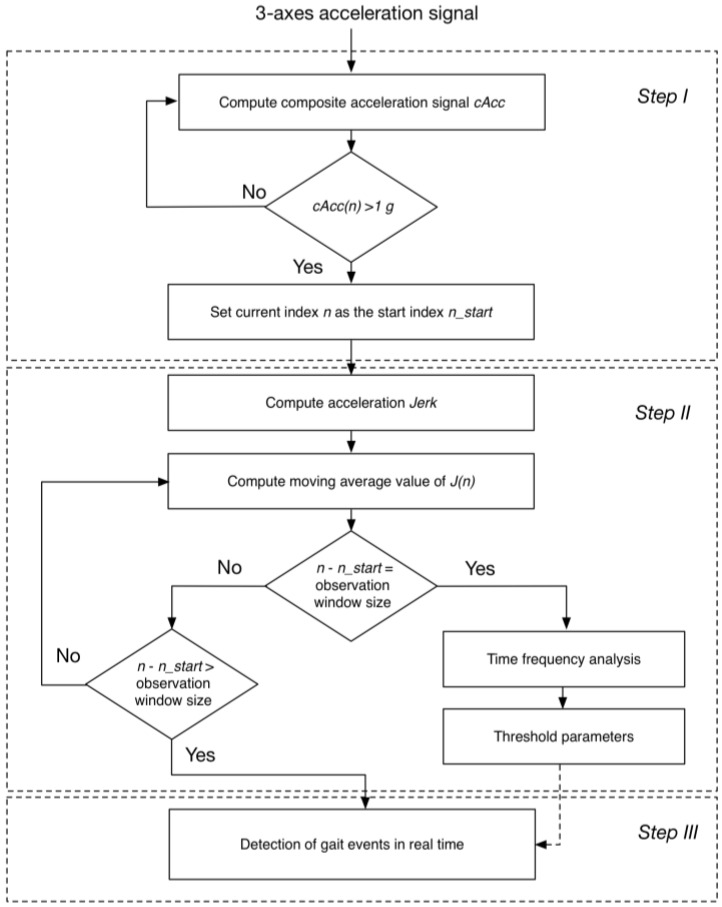
Flowchart of the proposed algorithm of gait event detection which consists of three main steps: (**I**) identification of the transition from rest to walking; (**II**) determination of the thresholds for real-time heuristic algorithm based on time-frequency analysis; and (**III**) detection of gait events in real-time.

The proposed algorithm consists of three main steps: (1) identification of the transition from rest state to walking; (2) determination of the thresholds for the real-time heuristic algorithm based on the time-frequency analysis; and (3) detection of gait events in real-time, as shown in Figure 3. Each step of the algorithm is detailed in the following sections.

#### 2.4.1. Identification of the Transition from Rest to Walking 

In this stage, the composite acceleration signal was computed from the 3-axis acceleration recordings of each subject in each experimental trial and was then used as criteria to identify the transition procedure from rest to walking. The time of the identified transition onset serves as an input (starting point of the observation window) in the next step. The composite acceleration was computed by using the following equation.

(1) $$cAcc=\sqrt{{a_{x}}^{2}+{a_{y}}^{2}+{a_{z}}^{2}}$$

where *a_x_*, *a_y_*, and *a_z_* represent the acceleration signals recorded along the *x*, *y*, and *z* axes, respectively. When the value of *cAcc* is above a certain threshold denoted as *TH*_1_, the time index of the current point is referred to as the starting point (*n_start*). The threshold *TH*_1_ was set to a sufficiently large value (1*g N/m, where g denotes the acceleration due to gravity) to ensure accurate identification of the walking state. The differential acceleration (*Jerk*) was computed using Equation (2).

(2) $$Jerk=\sqrt{{\left(\frac{da_{x}}{dt}\right)}^{2}+{\left(\frac{da_{y}}{dt}\right)}^{2}+{\left(\frac{da_{z}}{dt}\right)}^{2}}$$

Furthermore, to remove some small peaks of the *Jerk* signals for easy detection of the gait events, the moving average value of the *Jerk* was calculated to obtain the smoothed data *J(n)*, as described in the following equation:

(3) $$J(n)=\frac{\sum_{n-m-1}^{n}Jerk(n)}{m}$$

where *m* and *n* denote the size of the sliding window and the sample number, respectively. In selecting a value for *m*, a trade-off between time latency and smoothing effects should be considered. In this study the value of *m* was set to 30 data samples. The smoothed data *J(n)* was used for the time-frequency analysis in the observation window and for the gait event identification.

#### 2.4.2. Determination of the Thresholds for the Real-Time Heuristic Algorithm Based on the Time-Frequency Analysis

For each subject, the determination of the thresholds for the real-time heuristic based algorithm was performed on an observation window that provided an efficient means to analyze data samples with a predefined length. It also served as a window to explore the inherent gait characteristics such as swing time, stance time, and peak amplitude of gait events. Then, these gait characteristics could be used as domain knowledge to specify reliable threshold values for heuristic based real-time gait event detection. 

Time-frequency analysis approaches offer an interpretation of a signal in both the time and frequency domains simultaneously, from which local, transient, and intermittent components of the signal can be elucidated [26]. Continuous wavelet transform (CWT) is a widely used time-frequency analysis tool that effectively captures the general characteristics of the signal under observation [27]. Furthermore, CWT with a proper mother wavelet has shown robustness in detecting gait events [20,27,28,29]. The Morlet mother wavelet was adopted in this study to investigate the time-frequency relationship between the gait event and gait cycle [20]. The fundamental knowledge of the Morlet wavelet function and CWT are described as follows:

(4) $$\psi_{0}(\eta )=\frac{1}{\sqrt[{4}]{\pi }}e^{iw_{0}\eta }e^{-\frac{\eta^{2}}{2}}$$

The CWT of a discrete time signal (*x_n_*), with equal time spacing (δ*_t_*), is defined as the inner product of *x_n_* with a scaled and translated Morlet mother wavelet ψ_0_(η).

(5) $$W_{n}(s)=\sum_{n^{\prime }=0}^{N-1}x_{n^{\prime }}\psi *[\frac{(n^{\prime }-n)\delta_{t}}{s}]$$

where *W_n_(s)* denotes the wavelet transform, *s* is the wavelet scaling factor, *n* is the localized time index, and the (*) indicates the complex conjugate. The frequency scale relationship of the wavelet can be represented as shown in Equation (6).

(6) $$f=\frac{f_{c}\times F_{s}}{s}$$

where *f_c_* is the central frequency of the wavelet and *F_s_* is the data sampling frequency. The central frequency of the Morlet wavelet was chosen to be 0.8125 Hz and the *F_s_* was 148.15 Hz. The minimum gait frequency was assumed to be 0.5 Hz and the corresponding maximum scale denoted as *s_max_* for the analysis was set to 241. 

**Figure 4:**
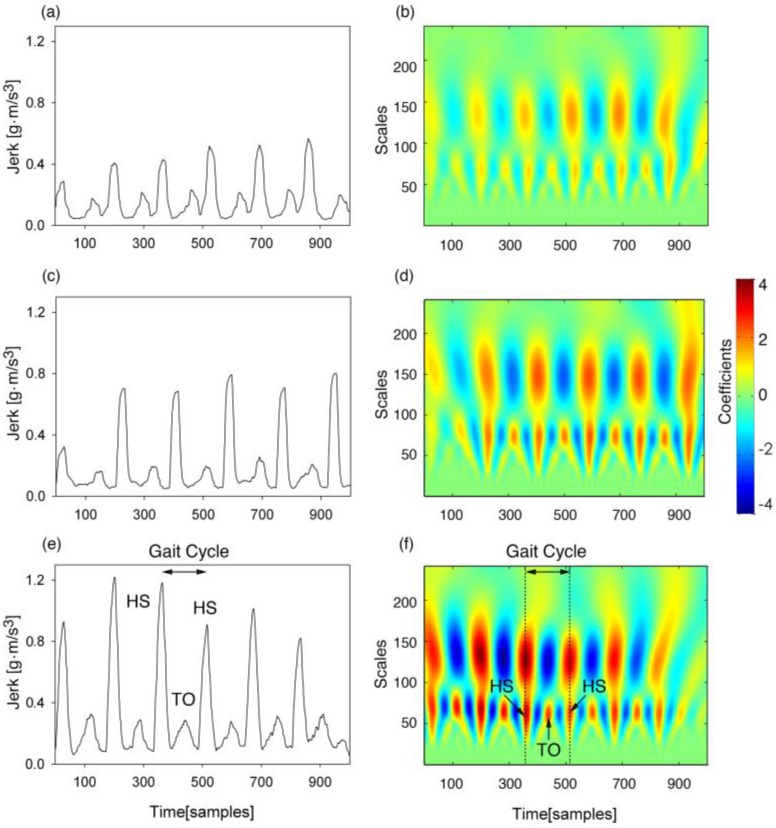
Illustration of the smoothed acceleration *Jerk* signals on the three terrains for a subject (left panel) and their corresponding time-frequency representation based on the Morlet wavelet (right panel). The individual gait events of heel strike (HS) and toe off (TO) can be observed in the continuous wavelet transform (CWT) coefficients. The results from the top row to the bottom row are from the terrains of level ground (**a**,**b**), up stairs (**c**,**d**), and down stairs (**e**,**f**), respectively.

The typical smoothed *Jerk* signals *J(n)* and their corresponding CWT time-frequency signals are represented in Figure 3, for when a subject was walking on the three terrains. It can be observed from Figure 3 that the amplitudes of smoothed *Jerk* signals are significantly different across the three terrains. Thus it would be very difficult to use a given threshold on the amplitudes of the *J(n)* signals for the real-time detection of gait events when walking on different terrains. From Figure 4, we can also see that the time-frequency relationship between the individual gait events (HS and TO) and their corresponding gait cycleTable is represented clearly. Therefore, it would be possible to use the time-frequency characteristics of acceleration signals to capture gait events during walking as reported in a previous study [20]. Hence, we applied the time-frequency analysis technique to the data in the observation window to detect gait events and also obtained the desired threshold parameters for the real-time heuristic based algorithm. 

**Figure 5:**
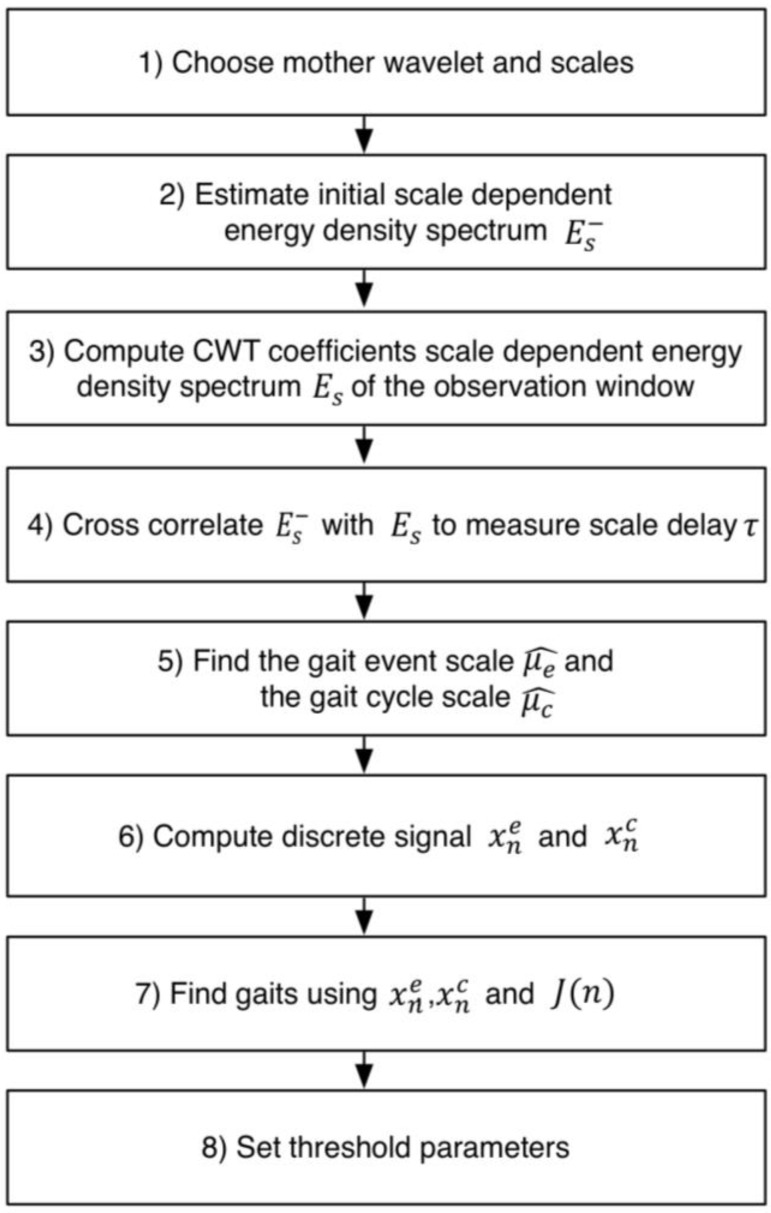
Flowchart of the time-frequency analysis of data in the observation window. Gait parameters can be obtained from the observation window to develop heuristics for real-time gait event detection. $E_{s}^{-}$ is the prior energy density spectrum estimate and the scale dependent energy density spectrum of the observation window. ${\hat{\mu }}_{e}$ and ${\hat{\mu }}_{c}$ denote the obtained scales of the gait event and the gait cycle. The corresponding CWT coefficients of these scales are denoted by *x*_e_ and *x*_c_.

The flowchart of the time-frequency analysis, gait event detection, and threshold parameter setting as applied in this study is represented in Figure 5. Each step in the figure is described as follows:

Step 1: The Morlet wavelet was chosen as the mother wavelet function, and the maximum scale of this function was set to 241 as previously described. 

**Figure 6:**
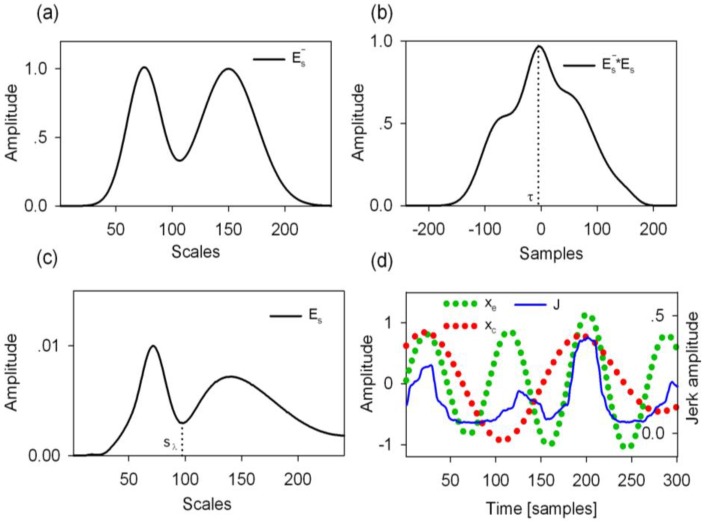
Graphical illustration of the gait event detection procedure in one observation window. (**a**) Initially estimated scale dependent energy spectrum $E_{s}^{-}$; (**b**) The cross correlation of the prior estimate $E_{s}^{-}$ with the current scale dependent energy spectrum $E_{s}$ to measure the scale delay τ between them; (**c**) The current scale dependent energy spectrum $E_{s}$ was used to find the scales of the gait event and the gait cycle; (**d**) The obtained CWT coefficients of *x*_e_ and *x*_c_ were used to determine the peaks in the smoothed *Jerk* signal *J*(*n*) for gait event detection.

Step 2: Initially, the frequencies of the gait events and cycle events were assumed to be 1.6 Hz and 0.8 Hz, respectively. Thus, the corresponding scales of the gait events and gait cycles were 75 and 150, respectively. For simplicity, the prior energy density spectrum estimate $E_{s}^{-}$ was approximated as a mixture of two one-dimension (1-D) Gaussian distributions based on Equation (7).

(7) $$E_{s}^{-}=e^{-{\left(\frac{s-\mu_{e}^{-}}{\sigma_{1}^{-}}\right)}^{2}}+e^{-{\left(\frac{s-\mu_{c}^{-}}{\sigma_{2}^{-}}\right)}^{2}}$$

where $E_{s}^{-}$ is the prior energy spectrum density estimate, $\mu_{e}^{-}$ is the prior scale of the gait event, $\mu_{c}^{-}$ is the prior scale of the gait cycle, and $\sigma_{1}^{-}$ and $\sigma_{2}^{-}$ denote the standard deviations. The values of $\mu_{e}^{-}$, $\mu_{c}^{-}$, $\sigma_{1}^{-}$, and $\sigma_{2}^{-}$ were 75, 150, 15, and 25, respectively. The detailed illustration of $E_{s}^{-}$ is graphically shown in Figure 6a.

Step 3: In each observation window, the CWT coefficients were computed from the input smoothed *Jerk* data using the pre-defined Morlet wavelet and scales. The size of the observation window was set to be longer than the longest duration of one gait cycle. In the study, the observation window was made to contain 300 sample points which were approximately two seconds in duration. Then, the scale dependent energy density spectrum *E_s_* was computed using Equation 8.

(8) $$E_{s}=\sum_{n=0}^{N-1}|W_{n}(s){|}^{2},s\in [1,s_{\max}]$$

where |*W_n_(s)*|^2^ is the 2-D wavelet energy density function that measures the scale dependent total energy distribution of a signal, *E_S_* is described in Figure 6c, and *s_max_* represents the maximum scale.

Step 4: The cross correlation between the prior estimate $E_{s}^{-}$ and *E_s_* was calculated to measure the scale delay τ (see Figure 6b) which was obtained using Equation (9).

(9) $$\tau =\arg\max_{s\in [1,s_{\max}]}(E_{s}^{-}*E_{s})$$

Step 5: After the scale delay τ was determined, the scale s_λ_ could be computed based on the mathematical expression in Equation (10).

(10) $$s_{\lambda }=\arg\min E_{s}$$

where $s\in [\mu_{e}^{-}+\tau ,\mu_{c}^{-}+\tau ]$ and s_λ_ was the scale where *E_s_* had a minimum value (See Figure 6c). The s_λ_ was used to distinguish the scale range of the gait event and the gait cycle. Thus, the posterior gait event scale ${\hat{\mu }}_{e}$ and the gait cycle scale ${\hat{\mu }}_{c}$ were later obtained using Equations (11) and (12), respectively.

(11) $${\hat{\mu }}_{e}=\arg\max_{s\in [1,s_{\lambda }]}E_{s}$$

(12) $${\hat{\mu }}_{c}=\arg\max_{s\in [s_{\lambda },s_{\max}]}E_{s}$$

Step 6: With the event scale ${\hat{\mu }}_{e}$ and the cycle scale ${\hat{\mu }}_{c}$, the CWT coefficients of these scales were determined and labeled as *x_e_* and *x_c_*, respectively. Furthermore, *x_e_* and *x_c_* were filtered using a second-order Butterworth high-pass filter (forward and backward) with a cutoff frequency of 10 Hz to remove high frequency noises.

Step 7: With these signals of *x_e_*, *x_c_*, and *J(n)*, the gait events could be identified, as shown in Figure 6d. The negative peaks of *x_e_* were recognized and their indices were used to divide the observation window into different event regions. Gait events were identified as the maximum value of *J(n)* in the region of the two neighboring negative peaks of *x_e_*. Also, the first event region was marked with the index range of [1, n_1stNP], where n_1stNP denotes the index of the first negative peak of *x_e_*. Similarly, the final gait event was identified within the index range of [n_lastNP, 300], where n_lastNP denotes the index of the last negative peak of *x_e_*. Meanwhile, the gait event (HS or TO) time was identified as the peak index of *J(n)* in the region under consideration. Subsequently, the gait event corresponding to an event region would be classified as HS when a positive peak of *x_c_* is observed, or TO when a negative peak of *x*_c_ is observed.

Step 8: The gait event (HS and TO) times and the corresponding amplitudes of *J(n)* within the observation window were stored and used to compute the threshold parameters. A baseline threshold (designated as *TH*_2_) was firstly defined as the median value of *J(n)* in the observation window for improving the reliability of gait event detections. Then, for the HS events within the observation window, their threshold value (designated as *TH*_3_) was defined as the average amplitude of the peaks of *J(n)* corresponding to HS events, and for the TO events, their threshold value (designated as *TH*_4_) was defined as that corresponding to TO events. The average swing phase time (*T_swin_)* and the average stance phase time (*T_std_*) were also defined. 

#### 2.4.3. Detection of Gait Events in Real-Time

The procedure of gait event detection during walking is described as follows:

Step 1: With the threshold values obtained above, the next task was to detect the peak of the current gait event from the signal *J*(*n*). When a peak was firstly detected from *J*(*n*) and its amplitude was greater than a given value that was set as r_b_ × *TH*_2_ (r_b_ = baseline membership), the peak was designated as *J*(*n_p_*) and was used as a starting point to search the potential peaks in Step 2.

Step 2: Wait a certain time with an attempt to check if the detected peak *J*(*n_p_*) is the real peak corresponding to a gait event. In the study, a 15 sample point waiting duration (about 100 ms) was chosen after the experimental investigations, which would efficiently avoid the false peaks and have a reasonable time latency. Note that a longer waiting duration should be desired for reliably obtaining the real peak, but would also increase the time latency of gait event detections. Then, check if there were peaks in the subsequent 15 samples of the signal *J*(*n*) that had an amplitude greater than *J*(*n_p_*). If there were, the peak with a maximum amplitude was used as the new starting point (*J*(*n_p_*)) and then this step was repeated. Otherwise, move to Step 3.

Step 3: Further check that the amplitude of the current peak *J*(*n_p_*) was greater than the amplitude of all the subsequent 15 samples. If it was, the peak was considered as the candidate peak (designated as *J*(*n_c_*)) and then proceed to next step. Otherwise, search for the subsequent peak that served as a new starting point (*J*(*n_p_*)) and repeat Step 2.

Step 4: If the previous gait event was TO, the candidate peak *J*(*n_c_*) was identified as an HS event when the amplitude of the candidate peak was greater than r_1_ × *TH*_3_ (r_1_ = stride amplitude membership) and the time interval between the last gait event (TO) and the candidate peak was greater than r_2_ × *T_swin_* (r_2_ = stride duration membership). If the previous gait event was HS, the candidate peak *J*(*n_c_*) was identified as a TO event when the amplitude of the candidate peak was greater than r_1_ × *TH*_4_ and the time interval between the last gait event (HS) and the candidate peak was greater than r_2_ × *T_std_*. Otherwise, the candidate peak would be discarded and Step 1 was repeated. 

### 2.5. Performance Evaluation of the Proposed Algorithm 

The performance of the proposed algorithms in identifying TO and HS gait events was evaluated in terms of the accuracy and timing agreement with respect to the FSR method. Walking data of the first 300 sample points (first few cycles) that served as an observation window were used for the time-frequency analysis and the data corresponding to the last gait cycle was excluded in the computation. The accuracy was assessed using Precision (P), Recall (R), and F1 score measures, which are defined in Equation (13), where TP denotes true positives, FN represents false negatives, and FP denotes false positives. In addition, TP represents the number of correctly detected gait events, FN denotes the number of missed gait events, and FP symbolizes the number of wrongly detected gait events.

(13) $$P=\frac{TP}{TP+FP},R=\frac{TP}{TP+FN},F1=\frac{2PR}{P+R}$$

In the study, a metric, the timing agreement between the proposed method and the FSR method, was adopted to measure the time difference for successfully detecting a walking event. A Bland-Altman plot was used to represent the timing agreement between the proposed method and the FSR method. In the FSR method, the HS and TO gait events were identified at 5% increase in the maximum heel FSR amplitude and 5% decrease in the maximum toe FSR amplitude, respectively. The maximum heel and toe FSR amplitudes were defined with respect to the FSR signal segments in the observation window and then the time instance of the correctly detected gait events of the proposed and FSR methods were compared. Additionally, the time of gait event detection was assessed for the proposed and FSR method using the absolute mean difference (AMD), mean difference (MD), and 95% confidence interval (CI) [30]. 

### 2.6. Parameter Selection 

**Figure 7:**
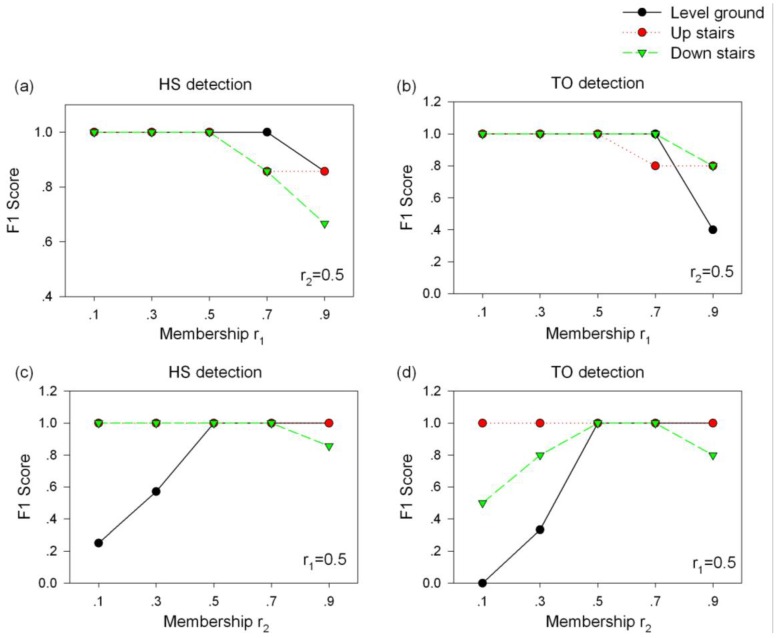
A plot of the F1 scores for gait event detection versus the membership of r_1_ and r_2_ on different terrains. r_1_ denotes the stride amplitude membership and r_2_ represents the stride duration membership. F1 scores were computed from a representative trial on each terrain from a subject.

The value of the membership *r_1_* and *r_2_* described above were selected according to the accuracy of the obtained F1 scores using one trial representative gait data on each terrain per subject. To obtain the values for the *r_1_* and *r_2_* parameters, we iteratively examined a set of values in the range of 0.1 to 0.9 (0.1, 0.3, 0.5, 0.7, and 0.9) and eventually realized that when *r_1_* and *r_2_* were both 0.5, the performance of the algorithm was relatively good and stable, as shown in Figure 7. Meanwhile, after inspecting the data across all the recruited subjects, it was observed that a baseline membership (*r_b_*) of 0.8 would have an insignificant effect on the performance of the proposed algorithm with respect to gait event detection.

## 3. Results

### 3.1. Gait Event Detection

**Figure 8:**
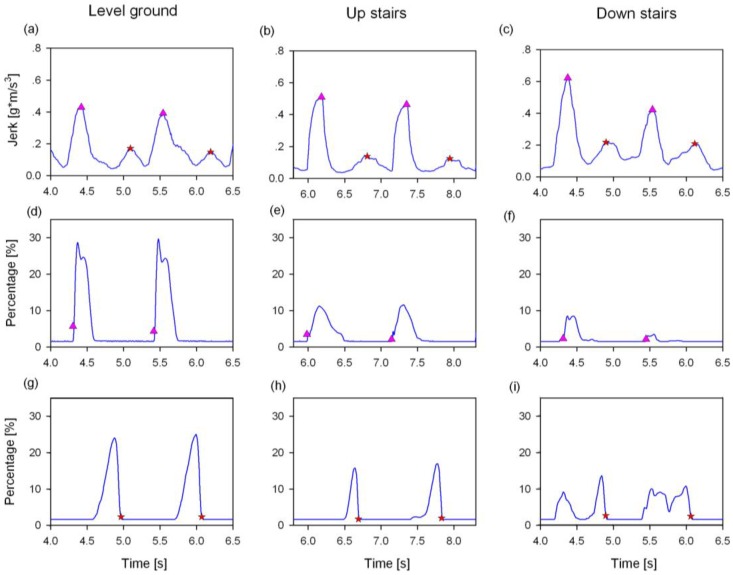
Typical results of the gait event detections on different terrains from one subject by using both the proposed and FSR based method. The heel strike (HS) and the toe off (TO) are marked by a pink triangle and a red star, respectively. The HS and TO events from the proposed algorithm are represented in (**a**–**c**) for the three terrains, respectively. The HS and TO events obtained by FSR method are illustrated in (**d**–**i**), respectively.

The typical results of the gait event detection based on the proposed algorithm and the FSR reference method are illustrated in Figure 8. It can be seen from Figure 8a–c that the smoothed acceleration *Jerk* signals were similar in shape when walking on the three terrains, which would make the detection of gait events easy and convenient. Compared to the proposed method, the FSR signals of both the HS and TO events changed considerably among the three terrains, as shown in Figure 8d–i. The instability of the FSR signals on different terrains would sometimes affect the accuracy in detecting a gait event. 

### 3.2. Accuracy of Gait Event Detection

The average accuracies (precision, recall, and F1 score) of both HS and TO event detection on different terrains over all eight subjects are summarized in Table 1 and Table 2, respectively. From Table 1 (HS event detection), we can see that the proposed algorithm achieved similar F1 scores as the conventional FSR method on the three terrains. Only for the down stairs terrain, the F1 score of the proposed algorithm was 0.98, which was slightly larger than that of the FSR method (0.97). From Table 2 (TO event detection), for level ground walking, the F1 score of the proposed algorithm was similar to that of the FSR method. It is important to note that for the walking on up and down stairs terrains, the F1 scores of the proposed algorithm were obviously higher than those of the FSR method. The proposed algorithm could achieve a F1 score of 0.99 and 0.98 for up stairs and down stairs, respectively, while the F1 scores of the FSR method were 0.92 and 0.94, respectively.

### 3.3. Timing Agreement between the Proposed Algorithm and FSR Method

**Figure 9:**
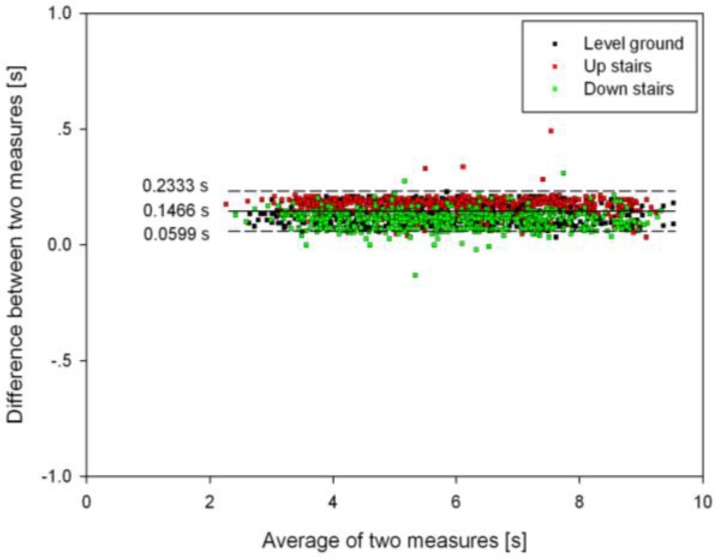
Bland-Altman plot of time agreement between the proposed algorithm and the FSR method for HS gait event detection on the three terrains. The horizontal axis is the average of the time measures of detecting gait events by both methods, and the vertical axis is the difference between the two time measures. Positive time differences represent a delay in the proposed algorithm with respect to the FSR method. The solid horizontal line denotes the mean error while the dashed horizontal line is the limits of time agreement (Mean + 1.96 SD).

**Figure 10:**
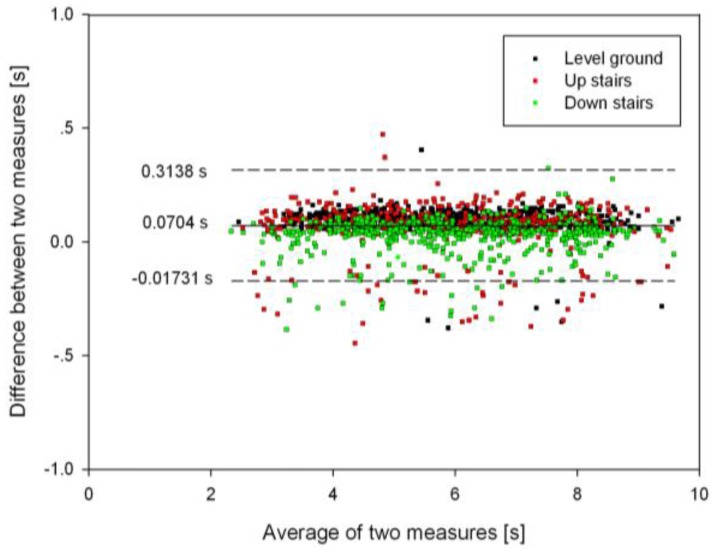
Bland-Altman plot of time agreement between the proposed algorithm and the FSR method for TO gait event detection on different terrains. Positive time differences correspond to a delay in the proposed algorithm with respect to the FSR method. The solid horizontal line denotes the mean error while the dashed horizontal lines represent the limits of timing agreement (Mean + 1.96 SD).

The Bland-Altman plot of the timing agreement between the proposed algorithm and the FSR method with respect to the detection of HS and TO gait events for the three terrains is shown in Figure 9 and Figure 10, respectively. Note that the positive values in the plot represent the delay in the proposed algorithm in comparison with the FSR method. From Figure 9 and Figure 10, it can be observed that the proposed algorithm attained an average timing delay of about 146.6 ms and 70.4 ms for HS and TO gait events, respectively. For HS event detection, the upper and lower limits of the timing agreement were 59.9 ms and 233.3 ms (Mean + 1.96 SD), and for TO event detection, the timing agreement limits were −173.1 ms and 313.8 ms (Mean + 1.96 SD). Note that the larger timing agreement limits observed for TO event detection were caused by the large time delays in detecting some gait events during stair walking (Figure 10). In addition, AMD, MD, and 95% CI of the gait event detection time on the three terrains between the proposed and FSR method are shown in Table 3. Again, a larger time delay could be observed for the HS events compared to that of the TO events. For HS gait events, AMD values were found to increase in the following order: down stairs (119.6 + 36.2 ms), level ground (137.4 + 41.2 ms), and up stairs (173.9 + 35.6 ms). For TO gait events, AMD values were observed to increase as follows: down stairs (72.2 + 57.1 ms), up stairs (116.2 + 80.6 ms), and level ground (124.4 + 132 ms). 

### 3.4. The Comparison of F1 Score with Previous Studies for Stair Terrain

In the study conducted by Kotiadis et al., an accelerometer based method was used for gait event detection on stair terrain, with one missed gait event and three wrongly detected gait events in a total of eleven gait events [2]. Based on these values, the F1 scores for the gait events of HS and TO were computed as reported in Table 4. Additionally, Formento et al. adopted a gyroscope based heuristic algorithm for gait event detection and reported that two TO gait events were missed in 20 TO events on up stairs terrain. Furthermore, three TO events were missed while one was wrongly detected in a total of 27 TO events on down stairs terrain [30]. With this data, the F1 score for their method was also computed and reported in Table 4. Subsequently, the F1 score obtained from our proposed algorithm was compared with those of Kotiadis et al. and Formento et al.

## 4. Discussion

The aim of this study was to develop and assess an accelerometer based algorithm for real-time gait event detection on different terrains. To assess the performance of the newly proposed algorithm in detecting gait events during walking, the conventional FSR based foot switches were used as a reference method. It was observed that the average F1 score for the FSR method across all recruited subjects were found to be 1.00 for both HS and TO gait event detection on level ground terrain. However, there was a decrease in the average F1 score obtained during gait event detection while walking on stair terrain in comparison to level ground walking. The difference in the F1 scores might be attributed to the instability noticed in the FSR signal recordings while walking up and down the stairs. Additionally, the procedure of putting on and taking off FSR sensors are usually complex and often introduce some level of discomfort and inconvenience to the wearers. These factors will eventually limit their applications in real life [14].

As an alternative to the FSR sensor for gait event detection, an accelerometer could provide reliable gait event signals, and it requires relatively low power consumption, is low cost, and is more convenient to wear [20]. The proposed acceleration based algorithm could detect gait events with robustness and accuracy in real-time applications. Our results showed that average F1 scores of 0.99 (up stairs) and 0.98 (down stairs) for HS gait event detection were recorded, while the average F1 scores of 0.95 (up stairs) and 0.99 (down stairs) for TO gait event detection were obtained. To the best of our knowledge, the gait event detection on stair terrains (ascending and descending stairs) has rarely been investigated. Kotiadis et al. proposed an acceleration based algorithm for gait event detection on different terrains. In their study, F1 scores of 0.96 and 0.76 for HS on up stairs and down stairs terrains were respectively obtained [2]. Formento et al. proposed a rate gyroscope based heuristic algorithm for gait event detection, and recorded F1 scores of 0.95 and 0.93 for HS on up stairs and down stairs terrains respectively [30]. A major limitation of their study was that very few gait cycles were considered. It is important to note that the F1 scores attained by the proposed algorithm is relatively higher than that of the previously proposed methods. Hence, our proposed algorithm may have the potential for accurate gait event detection on stair terrains in real-time applications in comparison to the previous methods. 

The mean time delay of the proposed method in comparison to the FSR method was about 146.6 ms for HS and 70.4 ms for TO. Compared to the delays reported in some previous studies [14,31], the increase in time delay especially for HS in our study may be attributed to the 30 point sliding window which was used to smoothen the acceleration *Jerk*. 

In the proposed algorithm, the size of the observation window was fixed and set to about two seconds. It should be noted that the correct detection of gait events relies on the gait events identified in the observation window using time-frequency analysis and the heuristics developed based on parameters obtained in the observation window. Thus the errors in detecting gait events may be due to the wrongly identified gait events in the observation window and/or the developed heuristics. Continuous wavelet transform could effectively capture gait events and cycles from the acceleration signal [20,32]. However, if there are sudden changes in gait speed, the time-frequency analysis method may not correctly capture all the gait events in the observation window, leading to incorrect threshold parameter estimation for the heuristic approach. Thus, the subjects were asked to walk in a comfortable way without sudden speed changes during the experiments. In the developed heuristics, gait amplitude parameters (0.5 × *TH*_3_, and 0.5 × *TH*_4_) and gait duration parameters (0.5 × *T_swin_* and 0.5 × *T_std_*) were computed to identify each type of gait event. These parameters yielded results that are relatively good and comparable to those reported in previous studies. However, the parameters could be further optimized especially when considering a tradeoff between gait event detection accuracy and robustness in real life applications. In addition, the proposed algorithm’s performance was only tested with data acquired from eight healthy subjects. Future work will include testing the proposed algorithm using datasets acquired from a number of pathological gait patients and making the necessary adaptations to the algorithm. 

## 5. Conclusions

This paper proposed a new algorithm to detect gait events on three walking terrains in real-time based on the analysis of acceleration jerk signals with a time-frequency method to obtain gait parameters, and to then determine the peaks of the jerk signals using peak heuristics. The accuracy and robustness of the algorithm were validated by using leg-acceleration signals from eight healthy subjects while walking on level ground, up stairs, and downs stairs terrain. The experimental results showed that the proposed algorithm can accurately detect toe off and heel strike gait events with comparable accuracy and time delays across different terrains. Testing and adapting the algorithm with varying speeds and pathological gait patients will be conducted in the future work. 

## Figures and Tables

**Table 1:** Performance of the proposed algorithm and FSR based method for HS gait event detection on the three terrains in terms of precision, recall, and F1 score (Mean ± SD) across all eight subjects.

Accuracy	Level Ground		Upstairs		Downstairs	
	ACC	FSR	ACC	FSR	ACC	FSR
P	1.00 ± 0.00	1.00 ± 0.00	0.99 ± 0.04	0.99 ± 0.08	0.98 ± 0.02	0.97 ± 0.03
R	0.99 ± 0.02	1.00 ± 0.00	0.99 ± 0.04	0.99 ± 0.02	0.98 ± 0.02	0.97 ± 0.11
F1	1.00 ± 0.01	1.00 ± 0.00	0.99 ± 0.04	0.99 ± 0.02	0.98 ± 0.02	0.97 ± 0.07

Note: And ACC denotes accelerometer.

**Table 2:** Performance of the proposed algorithm and FSR based method for TO gait event detection on the three terrains in terms of precision, recall, and F1 score (Mean ± SD) across all eight subjects.

Accuracy	Level Ground		Upstairs		Downstairs	
	ACC	FSR	ACC	FSR	ACC	FSR
P	0.99 ± 0.02	1.00 ± 0.01	0.95 ± 0.09	0.93 ± 0.08	0.99 ± 0.02	0.98 ± 0.03
R	0.98 ± 0.03	1.00 ± 0.01	0.95 ± 0.09	0.92 ± 0.08	0.99 ± 0.02	0.92 ± 0.03
F1	0.99 ± 0.02	1.00 ± 0.01	0.95 ± 0.09	0.92 ± 0.08	0.99 ± 0.02	0.94 ± 0.03

**Table 3:** Absolute mean difference (AMD), Mean Difference (MD), and 95% Confidence Interval (CI) in the timing of detected gait events on different terrains between the proposed algorithm and the FSR method.

Gait Event	Level Ground			Upstairs			Downstairs		
	AMD	MD	CI	AMD	MD	CI	AMD	MD	CI
HS	137.4 ± 41.2	137.4 ± 41.2	[133.6, 141.2]	173.9 ± 35.6	173.9 ± 35.6	[171, 176.8]	119.6 ± 36.2	119.5 ± 36.6	[116.1, 123.0]
TO	124.4 ± 132.0	115.8 ± 139.7	[102.8, 128.8]	116.2 ± 80.6	69.7 ± 123.1	[58, 81.4]	72.2 ± 57.1	26.4 ± 88.2	[18.3, 34.6]

Note: All results are expressed in milliseconds, while the average and standard deviation are reported for AMD and MD across all subjects.

**Table 4:** Comparison of the proposed algorithm and previously published results on stair walking based on F1 score.

	Sensor Type	Gait Events	F1 Score (Up Stairs)	F1 Score (Down Stairs)
This work	Acc	HS	0.99	0.98
This work	Acc	TO	0.95	0.99
[2]	Acc	HS	0.96	0.76
[2]	Gyro	HS	1	0.78
[30]	Gyro	TO	0.95	0.93

Note: Acc denotes accelerometer and Gyro denotes gyroscope.

## References

[1] Mansfield A., Lyons G.M. (2003). The use of accelerometry to detect heel contact events for use as a sensor in FES assisted walking. Med. Eng. Phys..

[2] Kotiadis D., Hermens H.J., Veltink P.H. (2010). Inertial gait phase detection for control of a drop foot stimulator. Med. Eng. Phys..

[3] Rueterbories J., Spaich E.G., Andersen O.K. (2014). Gait event detection for use in FES rehabilitation by radial and tangential foot accelerations. Med. Eng. Phys..

[4] Skelly M.M., Chizeck H.J. (2001). Real-time gait event detection for paraplegic FES walking. IEEE Trans. Neural Syst. Rehabil. Eng..

[5] Park Y.L., Chen B.R., Young D., Stirling L., Wood R.J., Goldfield E., Nagpal R. Bio-inspired active soft orthotic device for ankle foot pathologies. Proceedings of the 2011 IEEE/RSJ International Conference on Intelligent Robots and Systems.

[6] Lopez-Meyer P., Fulk G.D., Sazonov E.S. (2011). Automatic detection of temporal gait parameters in poststroke individuals. IEEE Trans. Inf. Technol. Biomed..

[7] El Achkar C.M., Lenoble-Hoskovec C., Paraschiv-Ionescu A., Major K., Büla C., Aminian K. (2016). Instrumented shoes for activity classification in the elderly. Gait Posture.

[8] Ghoussayni S., Stevens C., Durham S., Ewins D. (2004). Assessment and validation of a simple automated method for the detection of gait events and intervals. Gait Posture.

[9] Mills P.M., Barrett R.S., Morrison S. (2007). Agreement between footswitch and ground reaction force techniques for identifying gait events: Inter-session repeatability and the effect of walking speed. Gait Posture.

[10] Boutaayamou M., Schwartz C., Stamatakis J., Denoël V., Maquet D., Forthomme B., Croisier J.-L., Macq B., Verly J.G., Garraux G. (2015). Development and validation of an accelerometer-based method for quantifying gait events. Med. Eng. Phys..

[11] Rueterbories J., Spaich E.G., Larsen B., Andersen O.K. (2010). Methods for gait event detection and analysis in ambulatory systems. Med. Eng. Phys..

[12] Liberson W.T., Holmquest H.J., Scot D., Dow M. (1961). Functional electrotherapy: Stimulation of the peroneal nerve synchronized with the swing phase of the gait of hemiplegic patients. Arch. Phys. Med. Rehabil..

[13] Barrett C., Taylor P. (2010). The effects of the odstock drop foot stimulator on perceived quality of life for people with stroke and multiple sclerosis: Effectsofthe odstock drop foot stimulator. Neuromodulation.

[14] Bejarano N.C., Ambrosini E., Pedrocchi A., Ferrigno G., Monticone M., Ferrante S. (2015). A novel adaptive, real-time algorithm to detect gait events from wearable sensors. IEEE Trans. Neural Syst. Rehabil. Eng..

[15] González R.C., López A.M., Rodriguez-Uría J., Álvarez D., Alvarez J.C. (2010). Real-time gait event detection for normal subjects from lower trunk accelerations. Gait Posture.

[16] Lovse L., Bobet J., Roy F.D., Rolf R., Mushahwar V.K., Stein R.B. (2012). External sensors for detecting the activation and deactivation times of the major muscles used in walking. IEEE Trans. Neural Syst. Rehabil. Eng..

[17] Pappas I.P.I., Keller T., Mangold S., Popovic M., Dietz V., Morari M. (2004). A reliable gyroscope-based gait-phase detection sensor embedded in a shoe insole. IEEE Sens. J..

[18] Greene B.R., McGrath D., O’Neill R., O’Donovan K.J., Burns A., Caulfield B. (2010). An adaptive gyroscope-based algorithm for temporal gait analysis. Med. Biol. Eng. Comput..

[19] Bötzel K., Marti F.M., Rodríguez M.Á.C., Plate A., Vicente A.O. (2016). Gait recording with inertial sensors—How to determine initial and terminal contact. J. Biomech..

[20] Khandelwal S., Wickstrom N. (2016). Gait event detection in real-world environment for long-term applications: Incorporating domain knowledge into time-frequency analysis. IEEE Trans. Neural Syst. Rehabil. Eng..

[21] Sheehan R.C., Gottschall J.S. (2011). Stair walking transitions are an anticipation of the next stride. J. Electromyogr. Kinesiol..

[22] Au S., Berniker M., Herr H. (2008). Powered ankle-foot prosthesis to assist level-ground and stair-descent gaits. Neural Netw..

[23] Goršič M., Kamnik R., Ambrožič L., Vitiello N., Lefeber D., Pasquini G., Munih M. (2014). Online phase detection using wearable sensors for walking with a robotic prosthesis. Sensors.

[24] Sant’Anna A., Wickström N. (2010). A symbol-based approach to gait analysis from acceleration signals: Identification and detection of gait events and a new measure of gait symmetry. IEEE Trans. Inf. Technol. Biomed..

[25] Similä H., Immonen M., Merilahti J., Petäkoski-Hult T. Gait analysis and estimation of changes in fall risk factors. Proceedings of the 2015 37th Annual International Conference of the IEEE Engineering in Medicine and Biology Society (EMBC).

[26] Addison P.S., Walker J., Guido R.C. (2009). Time—Frequency analysis of biosignals. IEEE Eng. Med. Biol. Mag..

[27] Aung M.S.H., Thies S.B., Kenney L.P.J., Howard D., Selles R.W., Findlow A.H., Goulermas J.Y. (2013). Automated detection of instantaneous gait events using time frequency analysis and manifold embedding. IEEE Trans. Neural Syst. Rehabil. Eng..

[28] Rezvanian S., Lockhart T. (2016). Towards real-time detection of freezing of gait using wavelet transform on wireless accelerometer data. Sensors.

[29] Khandelwal S., Wickström N. Identification of gait events using expert knowledge and continuous wavelet transform analysis. Proceedings of the International Conference on Bio-Inspired Systems and Signal Processing.

[30] Formento P., Acevedo R., Ghoussayni S., Ewins D. (2014). Gait event detection during stair walking using a rate gyroscope. Sensors.

[31] Catalfamo P., Ghoussayni S., Ewins D. (2010). Gait event detection on level ground and incline walking using a rate gyroscope. Sensors.

[32] Khandelwal S., Wickström N. Detecting gait events from outdoor accelerometer data for long-term and continuous monitoring applications. Proceedings of the 13th International Symposium on 3D Analysis of Human Movement (3D-AHM 2014).

